# Supplementary material for: Dual Modular Titanium Alloy Femoral Stem Failure Mechanisms and Suggested Clinical Approaches
Source: Materials (Basel). 2021 Jun 4;14(11):3078. doi: 10.3390/ma14113078 (PMC8200202; doi:10.3390/ma14113078)
Supplement: Supplementary file 1 [file materials-14-03078-s001.zip › materials-1222178-supplementary.pdf]

materials

Supplementary materials

Dual Modular Titanium Alloy Femoral Stem Failure Mechanisms and Suggested Clinical Approaches

Jan Zajc *, Andrej Moličnik and Samo Karl. Fokter

Department of Orthopaedic Surgery, University Medical Centre Maribor, 5 Ljubljanska Street, 2000 Maribor, Slovenia; andrej.molicnik@ukc-mb.si (A.M.); samo.fokter@ukc-mb.si (S.K.F.)
* Correspondence: jan.zajc@ukc-mb.si

Citation:, Zajc, J.; Moličnik, A.; Fokter, S.K. Dual Modular Titanium Alloy Femoral Stem Failure Mechanisms and Suggested Clinical Approaches. *Materials* **2021**, *14*, 3078. https://doi.org/10.3390/ma14113078

Academic Editor: Piotr Piszczek and Aleksandra Radtke

Received: 29 April 2021
Accepted: 2 June 2021
Published: 4 June 2021

Publisher's Note: MDPI stays neutral with regard to jurisdictional claims in published maps and institutional affiliations.

Copyright: © 2021 by the authors. Licensee MDPI, Basel, Switzerland. This article is an open access article distributed under the terms and conditions of the Creative Commons Attribution (CC BY) license (http://creativecommons.org/licenses/by/4.0/).

1. Identification of Modular Neck Fracture Reasons.

Table S1. Identification of modular neck fracture reasons.

Modular Neck Material*	Number of Failed Implants	Reasons for Fracture		Time Since Primary Implantation	Neck Length and Orientation	Implant Producer, Model	Main Study Conclusions	Source No.
		Risk Factors	Mechanisms					
Ti6Al4V	1	Young age, high BMI, high activity level	Crevice corrosion, Traumatic event, Transition from tensile to shear mode of fracture	1.8 years	Long, straight	Wright, Profemur Z	Additional mechanical junctions can lead to corrosion processes resulting in fracture. There is a risk of implant fracture at the stem-neck junction when double-modular devices are used, particularly when those with a long neck are implanted in heavy patients.	9
Ti6Al4V	1	High BMI	Fretting corrosion, Pitting corrosion	4 years	Long, varus, AV 8°	Wright, Profemur Z	The lever arm, combined with the height and weight of the patient, probably resulted in increased stresses at the neck-stem modular junction.	10
Ti6Al4V	1	High BMI	NA	12 years	Long, straight	Cremascoli, GSP	Constant evaluation of laboratory material should be continued, even though it does not always guarantee proper information regarding *in*	11

							vivo parameters.	
Ti6Al4V	1	Large-diameter femoral head, long modular neck, M-o-M articulation, patient size, activity level	Crevice corrosion, Fretting corrosion	5 years	Long, varus, AV 8°	Wright, Profemur Z	The combination of large femoral head, long modular necks, metal-on-metal components, patient obesity, and activity level may produce the "perfect storm" for fretting, corrosion, and failure.	12
CoCr	1	NA	Selective crevice corrosion favouring multiple nucleation sites, Fatigue degradation, Heavy chemical attack, Intergranular corrosion Critical galvanic coupling	3.8 years	Long, varus	Wright, Profemur Gladiator plasma	The presence of the cathodic carbides favours the selective dissolution of the metal matrix around them generating an intergranular-like corrosion phenomenon.<br><br>The mechano-chemical fatigue phenomenon locally decreased the alloy fatigue limit to about 40% with respect to the limit of the same alloy without any surface defect triggered by corrosion.	15
Ti6Al4V	6	High BMI, younger age	Thin crack separating fatigue zone from the rapid fracture zone, A-lamellae	5.6 years (mean, range (2.3–12 years)	4 Long, straight, 2 Long, varus	2 Cremascoli, GSP<br>4<br>Wright, Profemur Z	Surface of our investigated modular femoral neck had a look of valleys and peaks with some µm of distance in	18

							between.<br><br>The best way of avoiding the complication of neck fracture is to refrain from using fully modular stems in primary THA.	
Ti6Al4V	7	High BMI, large total offset, large head diameter	NA	NA	Long	6 Wright, Profemur Z<br>1 Wright, Profemur E	85% of the arthroplasties used the same short, varus, retroverted neck; this same position could have been achieved with a non-modular neck.<br><br>The bimodular neck is unnecessary for routine use.	19
4 CoCr,<br>19 Ti6Al4V	23	Time since implantation, younger age, long necks, CoCr necks, male sex–	NA	CoCr: 3.4 years (SD ± 1.4),<br><br>Ti6Al4V: 5 years (SD ± 2.3)	19 Long,<br>4 Short	Wright, Profemur Z	Stem-neck modularity has no role in routine primary hip arthroplasty until issues with corrosion-induced fractures are resolved.<br><br>Further research of titanium-based alloys and design improvements for the manufacturing of bimodular stems is needed.	22
Ti6Al4V	1	High BMI, large femoral head, M-o-M bear-	NA	3.2 years	NA	Wright, Profemur Z	Findings at revision surgery were notable for	25

		ing					severe corrosion at the modular neck–body interface.<br><br>The use of stem designs that feature a modular neck as this additional interface creates a site for unique failure mechanisms that do not occur with monolithic stem designs.	
Ti6Al4V	1	High BMI	Fretting corrosion, Crevice corrosion, Microparticles release	7.8 years	Long, straight	Wright, Profemur Z	Patients with higher body mass index treated with long neck modular titanium-alloy THA may be at an increased risk to experience catastrophic failure of the device.<br><br>No reliable tool is currently available to predict the modular femoral neck fracture.	30
Ti6Al4V	1	−3.5 mm (S) head was exchanged with +7.0 mm (XL) head at acetabulum revision	NA	10 years (2 years after acetabulum revision)	Long, varus	Wright, Profemur Z	Corrosion resistance at the neck–taper interface should be substantially improved.<br><br>Regulators should change or accordingly adapt the testing protocols.	42

							Dual-modular stems for primary THA should be abandoned to avoid unnecessary complications.	
Ti6Al4V	2	Larger offset (long neck with + 3.5 mm (L) head), high BMI, younger age, male sex	NA	Left side: 3 years, right side: 20 years	Long, straight	Cremascoli, A.n.C.A Fit	Continued usage of bi-modular stems for primary THA is not only a problem associated with more treatment complications and patient suffering but also an economic problem and a threatening public health concern.	43
Average time for failure	/	/	/	5.8 years for Ti6Al4V necks, 3.5 years for CoCr necks	/	/	/	/
Σ	46	/	/	/	/	/	/	/

*All stems were made from Ti6Al4V alloy.

AV = Ante/Retro version; BMI–Body Mass Index; M-o-M – Metal-on-Metal; NA = Not Available; Wright = Wright Medical Technology Inc., Arlington, TN, USA; Cremascoli = Cremascoli S.p.A., Milan, Italy.
